# The Influence of the Vanishing Twin on the Perinatal Outcome of Surviving Singleton in IVF Pregnancy

**DOI:** 10.3389/fendo.2022.832665

**Published:** 2022-03-15

**Authors:** Jiarong Li, Jingyu Li, Yiyuan Zhang, Kuona Hu, Na Chen, Jie Gao, Jingmei Hu, Linlin Cui, Zi-Jiang Chen

**Affiliations:** ^1^ Center for Reproductive Medicine, Cheeloo College of Medicine, Shandong University, Jinan, China; ^2^ Research Unit of Gametogenesis and Health of Assisted Reproductive Technology (ART)-Offspring, Chinese Academy of Medical Sciences, Jinan, China; ^3^ Key Laboratory of Reproductive Endocrinology of Ministry of Education, Shandong University, Jinan, China; ^4^ Shandong Key Laboratory of Reproductive Medicine, Jinan, China; ^5^ Shandong Provincial Clinical Research Center for Reproductive Health, Jinan, China; ^6^ National Research Center for Assisted Reproductive Technology and Reproductive Genetics, Shandong University, Jinan, China; ^7^ Center for Reproductive Medicine, The Second Hospital, Cheeloo College of Medicine, Shandong University, Jinan, China; ^8^ Center for Reproductive Medicine, Ren Ji Hospital, School of Medicine, Shanghai Jiao Tong University, Shanghai, China; ^9^ Shanghai Key Laboratory for Assisted Reproduction and Reproductive Genetics, Shanghai, China

**Keywords:** *in vitro* fertilization - pregnancy, vanishing twin, perinatal outcome, assisted reproductive technology (ART), offspring

## Abstract

**Objective:**

The purpose of this study was to clarify the influence of the vanishing twin (VT) on the perinatal outcomes in the surviving singleton and further identify the susceptible window.

**Study design:**

Retrospective cohort study.

**Methods:**

A total of 636 survivors of a vanished co-twin and 11,148 singleton controls were enrolled. The exposed group was further divided into early VT (EVT, VT ≤13 weeks, N = 593) and late VT subgroups (LVT, VT >13 weeks, N = 43) according to the gestational age of the twin vanishing. All participants were conceived through *in vitro* fertilization (IVF). Perinatal outcomes including gestational age, birthweight, and the incidence of preterm birth (PTB), low birthweight (LBW), small for gestational age (SGA), neonatal intensive care unit (NICU) admission, umbilical cord abnormality, jaundice of the newborn, and oligohydramnios were compared among the groups.

**Results:**

In our birth cohort, about 5.4% of all singleton deliveries originated from vanishing twin pregnancies. Compared with the singletons, both early and late VT pregnancy had a significantly lower birth weight (3337.57±532.24 g and 2916.05±526.07 g vs. 3446.15±526.07 g; p < 0.001 and p < 0.001), more frequent neonatal jaundice (47.0% and 60.5% vs. 40.6%; p = 0.002 and p = 0.008), and decreased incidence of umbilical cord abnormality (15.5% and 7.0% vs. 19.9%; p = 0.009 and p = 0.034). Newborns in the early VT group were more likely to manifest as SGA (5.4% vs. 3.6%, p = 0.002) and suffered oligohydramnios (5.4% vs. 3.4%; p = 0.008) than the primary singletons. In addition, the gestational age of late VT survivors was shorter than that of the controls (37.25 ± 3.25 vs. 39.04 ± 1.63, p = 0.001) and had a significantly higher risk of PTB (30.2% vs. 6.6%; p < 0.001) and NICU admission (27.9% vs. 9.4%, p < 0.001). All differences except for SGA maintain significance after adjusting for maternal age, BMI, and parity.

**Conclusions:**

Singletons with a vanished co-twin had worse perinatal outcomes compared with the original singletons, with LVT burden even much on the survival one. Therefore, close monitoring during the perinatal period was suggested in this type of neonates. Moreover, elective single embryo transfer should also be fully considered which could tackle the problem at its root.

## Introduction

*In vitro* fertilization (IVF), as the final option or the sole solution for infertile couples, has led to more than 8 million births in the world (1). Singleton birth was the most common outcome after IVF treatment which possessed about 80% of all pregnancies (2). Yet, with the routine use of ultrasound scan in the first trimester and throughout the pregnancy period, it was found that not all singleton neonates originated from singleton pregnancies. Vanishing twin syndrome (VTS), defined as the spontaneous reduction of a twin fetus (3), was estimated to occur in 5%–30% IVF/intracytoplasmic sperm injection (ICSI) pregnancies (4, 5). This reduction could happen in the first trimester named as early vanishing twin (EVT) or in the late pregnancy trimester named as late vanishing twin (LVT) (6).

Different from the primary singletons, those born to VT pregnancies were exposed more or less to an intrauterine environment of twin pregnancy, which was supposed to be related to adverse pregnancy outcomes such as lower birthweight, premature birth (PTB), and even fetal and infant death (7–9). Besides, the pathophysiological response subsequent to the fetal miscarriage might bring additional risk to the surviving one (10). Previous studies reported that neonates with a co-VT twin showed lower birth weight and increased risk of PTB, small for gestational age (SGA), and birth defects compared with singleton pregnancies (3, 5, 6, 11), which may affect the long-term health of offspring with increased risks of depression and metabolic, cardiovascular, and renal complications (12, 13). However, some other studies did not find any evidence of the impact of VT on the survivors (14, 15). Heterogeneity of the fetal loss time and the underpower due to the limited samples might lead to contradictions in these studies.

The aim of the present study was to clarify the influence of twin vanishing on the perinatal outcomes of the survivors based on a retrospective cohort design in a large-scale population. Furthermore, subgroup analysis was performed according to the vanishing time to identify the susceptible window.

## Materials and Methods

### Study Design

The present study followed a retrospective cohort design. Singletons born after IVF from July 2014 to December 2017 were recruited at the Center for Reproductive Medicine, Cheeloo College of Medicine, Shandong University. The IVF procedures were described in our previous studies (16). After pregnancy diagnosis, the gestational sacs were monitored by three-dimensional (3D) ultrasound scan at 6–8 weeks and 11–13 weeks of gestation, respectively. Those with two gestational sacs but singleton birth were enrolled as the exposed group, while the single pregnancies with only one gestational sacs were set as the unexposed group. In order to identify the susceptible period, the exposed group were further divided into 2 subgroups named EVT (co-twin disappeared ≤13 gestational weeks) and LVT (co-twin disappeared>13 gestational weeks). Those with donor oocytes, donor sperm, unavailable records of ultrasound scan in the first trimester, and VTS of monozygotic twins were excluded. Finally, a total of 11,784 neonates were enrolled with 636 subjects in the exposed group (593 in EVT and 43 in LVT) and 11,148 singletons in the unexposed group.

### Measurements

Baseline characteristics referred to maternal factors such as age, body mass index (BMI), parity, infertility cause, and the diagnosis of gestational diabetes mellitus (GDM) and gestational hypertension. Perinatal outcomes included gestational age, birthweight, and the incidence of preterm birth (PTB), low birthweight (LBW), small for gestational age (SGA), neonatal intensive care unit (NICU) admission, umbilical cord abnormality, and oligohydramnios. PTB was defined as delivery before 37 weeks of gestation. Birth weight was recorded in grams, and low birth weight was defined as less than 2,500 g. SGA referred to the children with a birthweight below the lower 10th percentile for gestational age (17). Umbilical cord abnormality included too short, intertwin, kinking, torsion, abnormal cord insertion, and single umbilical artery.

### Statistical Analysis

All statistical analyses were performed using the SPSS 25.0 statistical software. The cohort characteristics were described using the percentiles (numbers) for categorical variables, while continuous variables were expressed as means ± SD. The unpaired t test was used to analyze the distribution of continuous variables. Categorical variables were compared by the chi-square test. Logistic regression analysis was performed to adjust the confounders, including maternal age, BMI, types of embryo transferred and parity. Effects were described as odds ratio (OR) for logistic regressions with 95% confidence intervals (CIs). p-value <0.05 was considered as statistically significant.

## Results

In our study cohort, 5.40% (636/11,784) of the singletons born through IVF were from VT pregnancies, among whom 93.24% (593/636) belonged to EVT and 6.76% (43/636) belonged to LVT. Maternal characteristics are presented in **Table 1**. No significant differences were observed in maternal age, BMI, gestational hypertensive diseases, GDM, and infertility-caused distribution among the three groups. The rate of frozen-thawed embryo transfer is significantly higher in the singleton group than in the EVT and LVT groups (56.7% vs. 32.4% and 18.6%, p < 0.001 and p < 0.001). Mothers with EVT were more likely to be nulliparous (84.1% vs. 76.8%; p < 0.001) and with anovulatory disorders (8.9% vs. 6.5%, p = 0.022) compared to women with singletons.

**Table 1:** Maternal characteristics of vanishing twins and singletons.

Characteristic	Singletons	EVT	LVT	p-value	p_EVT vs. Singletons_	p_LVT vs. Singletons_
N	11,148	593	43			
Maternal age (years)	31.81 ± 4.20	31.54 ± 4.22	30.84 ± 3.258	0.110	0.138	0.131
BMI (kg/m^2^)	23.45 ± 3.61	23.53 ± 3.61	23.19 ± 3.11	0.800	0.642	0.636
Nulliparous [% (n)]	76.8 (8551)	84.1 (499)	79.1 (34)	**<0.001**	**<0.001**	0.723
Frozen-thawed embryo [% (n)]	56.7 (6318)	32.4 (192)	18.6 (8)	**<0.001**	**<0.001**	**<0.001**
Infertility cause						
Tubal [% (n)]	87.6 (9699)	87.1 (507)	88.4 (38)	0.932	0.733	0.877
Anovulatory [% (n)]	6.5 (721)	8.9 (52)	4.7 (2)	0.063	0.022	0.625
Endometriosis [% (n)]	3.5 (389)	4.0 (23)	0 (0)	0.389	0.575	0.212
Male factor [% (n)]	10.5 (1159)	11.3 (66)	7.0 (3)	0.601	0.502	0.456
Maternal diseases during pregnancy						
Gestational hypertensive diseases [% (n)]	5.6 (627)	3.9 (23)	7.0 (3)	0.941	0.069	0.701
GDM [% (n)]	7.9 (877)	7.9 (47)	9.3 (4)	0.177	0.966	0.729

Data were shown as mean ± SD or percentage (number). Indicators with statistically significant differences are expressed in bold (p < 0.05).

EVT, early vanishing twin; LVT, late vanishing twin; BMI, body mass index; GDM, gestational diabetes mellitus.

Compared to the primary singletons, survivors exposed to either EVT or LVT showed significantly lower birth weight (3337.57±532.24 g and 2916.05±526.07 g vs. 3446.15±526.07 g; p < 0.001 and p < 0.001), increased LBW (5.4% and 25.6% vs. 3.4%; p = 0.012 and p < 0.001), and jaundice of the newborn (47.0% and 60.5% vs. 40.6%; p = 0.002 and p = 0.008), as well as decreased umbilical cord abnormality (15.5% and 7.0% vs. 19.9%; p = 0.009 and p = 0.034). In addition, only newborns in the EVT group had higher incidence of SGA (5.4% vs. 3.6%, p = 0.002) and oligohydramnios (5.4% vs. 3.4%; p = 0.008), while only those in the LVT group had shorter gestation age (37.25 ± 3.25 vs. 39.04 ± 1.63, p = 0.001) and higher rate of PTB (30.2% vs. 6.6%; p < 0.001) and NICU admission (27.9% vs. 9.4%, p < 0.001) (**Table 2**).

**Table 2:** Perinatal outcomes of singleton, EVT, and LVT births.

Outcome	Singletons	EVT	LVT	p-value	p_EVT vs. Singletons_	p_LVT vs. Singletons_
Sex, boys [% (n)]	53.8 (5994)	53.0 (314)	48.8 (21)	0.749	0.688	0.515
Gestational age (weeks)	39.04 ± 1.63	38.91 ± 1.76	37.25 ± 3.25	**<0.001**	0.068	**0.001**
PTB [% (n)]	6.6 (735)	8.3 (49)	30.2 (13)	**<0.001**	0.111	**<0.001**
Birth weight (g)	3446.15 ± 526.07	3337.57 ± 532.24	2916.05 ± 526.07	**<0.001**	**<0.001**	**<0.001**
LBW [% (n)]	3.4 (383)	5.4 (32)	25.6 (11)	**<0.001**	**0.012**	**<0.001**
SGA [% (n)]	3.6 (400)	5.4 (32)	7.0 (3)	**0.039**	**0.022**	0.236
Neonatal diseases						
Umbilical cord abnormality [% (n)]	19.9 (2215)	15.5 (92)	7.0 (3)	**0.004**	**0.009**	**0.034**
Jaundice of the newborn [% (n)]	40.6 (4530)	47.0 (279)	60.5 (26)	**<0.001**	**0.002**	**0.008**
NICU admission [% (n)]	9.4 (1043)	10.6 (63)	27.9 (12)	**<0.001**	0.303	**<0.001**
Oligohydramnios [% (n)]	3.4 (374)	5.4 (32)	2.3 (1)	**0.027**	**0.008**	0.708

Data were shown as mean ± SD or percentage (number). Indicators with statistically significant differences are expressed in bold (p < 0.05).

EVT, early vanishing twin; LVT, late vanishing twin; PTB, preterm birth; LBW, low birth weight; SGA, small for gestational age; NICU, neonatal intensive care unit.

The logistic regression was used to adjust for the effects of confounding factors such as maternal ages, BMI, types of embryo transferred, and parity, and the results are shown in **Table 3**. After adjustment, EVT was still associated with a higher risk of PTB (OR: 1.37, 95% CI: 1.00–1.86, p = 0.048), LBW (OR: 1.69, 95% CI: 1.16–2.46; p = 0.006), jaundice of the newborn (OR: 1.27, 95% CI: 1.07–1.50; p = 0.007), and oligohydramnios (OR: 1.70, 95% CI: 1.17–2.48; p = 0.006). Nevertheless, the rate of umbilical cord abnormality was significantly decreased in the EVT group (OR: 0.76, 95% CI: 0.61–0.97; p = 0.026). LVT was also associated with an even much higher risk of PTB (OR: 6.56, 95% CI: 3.31–12.99, p < 0.001), LBW (OR: 9.25, 95% CI: 4.48–19.08; p < 0.001), and jaundice of the newborn (OR: 2.15, 95% CI: 1.16–3.99.; p = 0.015), along with the increased risk of NICU admission (OR: 3.88, 95% CI: 1.93–7.78; p < 0.001).

**Table 3:** Logistic regression models of risk factors for adverse perinatal outcome.

Outcome	p-adjust _EVT vs. Singletons_	OR (95% CI)	p-adjust _LVT vs. singletons_	OR (95% CI)
Preterm birth	**0.048**	**1.37 (1.00,1.86)**	**<0.001**	**6.56 (3.31,12.99)**
LBW	**0.006**	**1.69 (1.16,2.46)**	**<0.001**	**9.25 (4.48,19.08)**
SGA	0.111	1.37 (0.93,2.02)	0.334	1.80 (0.55,5.89)
Jaundice of the newborn	**0.007**	**1.27 (1.07.1.50)**	**0.015**	**2.15 (1.16,3.99)**
Umbilical cord abnormality	**0.026**	**0.76 (0.61,0.97)**	0.069	0.34 (0.10,1.09)
NICU admission	0.184	1.21 (0.91,1.60)	**<0.001**	**3.88 (1.93,7.78)**
Oligohydramnios	**0.006**	**1.70 (1.17,2.48)**	0.740	0.71 (0.10,5.23)

p-adj: adjusted p value by maternal age, BMI, types of embryo transferred and parity using logistic regression. Indicators with statistically significant differences are expressed in bold (p < 0.05).

EVT, early vanishing twin; LVT, late vanishing twin; PTB, preterm birth; LBW, low birth weight; SGA, small for gestational age; NICU, neonatal intensive care unit.

## Discussion

The present study indicated that despite of singleton birth, survivors with a VT co-twin still had an increased risk of adverse perinatal outcomes. EVT would confer the risk of LBW, jaundice of the newborn, and oligohydramnios to the exposed fetus, while LVT would burden even more in severity expected for oligohydramnios. Additionally, it was associated with increased risks of PTB and NICU admission as well.

The prevalence of VT pregnancies in ART singleton births was reported within a rather wide range from 3.6% to 30% (4, 5, 18, 19). According to our data, about 5.4% of singleton births after IVF originated from VT pregnancies, which was similar to the prevalence of 5.8% reported by Shebl O et al. in Austria (5). Consistent with previous studies, our study also indicated that the vanishing happened mostly in the first trimester (20). Maternal nulliparity, ovulatory dysfunction was found to be associated with EVT incidence. The increased rate of nulliparity in the EVT group may indicate higher rates of primary infertility, possibly related to adverse factors of embryo implantation such as poor endometrial receptivity. In addition, anovulation is always accompanied by ovulation induction interventions. The role of ovulation induction treatment in embryo disappearance needs further study.

As expected, adverse perinatal outcomes including PTB, LBW, jaundice of the newborn, oligohydramnios, and NICU admission were found in VT singletons even after adjusting for maternal factors. Placenta dysfunction or abnormality was well accepted as the most important etiological factor. In VTS, the absorption of necrotic fetal placental tissue would result in increased release of proinflammatory cytokine and prostaglandin, subsequently initiating an inflammatory process (21, 22). In addition, placental abnormalities were considered to play an important role in poor perinatal outcomes. It has been shown that chronic placental inflammation (CPI) is related to preterm delivery, fetal growth restriction, and pregnancy loss (23). A mouse model confirmed that exposure to intrauterine inflammation leads to fetoplacental hemodynamic changes, increased cytokine/chemokine expression, and increased abortion rates (24). The absorption would also change the placental blood flow of the left one (14). The adaptive growth of the placenta to the uterine environment during early pregnancy may partly explain the changes in placenta anatomy in VTS (25). There are some results that showed that VTS-surviving fetuses have small placentas (14). Previous studies suggested that an abnormal placental structure would lead to impaired exchange capacity and increase the risk of fetal growth restriction (FGR) (26). This may be an adaptive mechanism by which the fetus slows its growth to reduce oxygen delivery.

Twin-to-twin transfusion syndrome (TTTS) is a serious condition that influences 10%–15% of monochorionic multiple pregnancies. The abnormal vascular connection within the placenta was a critical factor in the development of TTTS (27, 28). In untreated TTTS, the risk of perinatal mortality due to intrauterine fetal death, extreme preterm birth or miscarriage can be up to 80%–90% (29). Further studies were needed to confirm the role of TTTS in vanishing twin.

Intriguingly, the present study indicated that the later VTS occurred, the worse the perinatal outcomes manifested. It was consistent with a previous study generated from a Danish cohort which indicated that the NICU admission and mortality rates were significantly higher in the late (>22 weeks) and intermediate (8–22 weeks) vanish groups compared with the early vanish group (<8 weeks) (30). This further supported the above hypothesis of mechanism, causing LVT to lead obviously to more placental tissue absorption. Yet, it still needed clinical studies to verify the findings and basic scientific studies to explore the mechanism.

A recent meta-analysis found that singletons conceived from frozen-thawed embryos have a lower rate of PTB (RR: 0.90, 95% CI: 0.84–0.97) and LBW (RR: 0.72, 95% CI: 0.67–0.77) than those conceived from fresh embryo transfer (31). Yet in the present study, the differences between groups remained significant even after adjusting for types of embryo transferred, which indicated an independent risk contributed by VT. Besides, the increased risk of some neonatal diseases, such as jaundice of the newborn, which were not reported to be associated with FET needs more attention

The present study demonstrated the association of VTS and poor perinatal outcomes in the survivors based on a large-scale cohort and further identified the medium and late pregnancies as a more susceptible window. The result would be an important indication for women with VTS in perinatal management. It also provided a supportive evidence for elective single-embryo transfer during ART treatment. Yet, our study also had some limitations. First, the study was based on a retrospective study design, which could not avoid its inherited bias. Second, the number of samples in the late disappearance group is relatively small, which would limit the statistical power. Third, the results were obtained from an IVF population, which might be inconsistent with natural conceived women. However, considering the prevalence of VT in the IVF population, the clinical management was more urgent to be improved for them.

## Conclusion

In conclusion, despite of singleton birth, women with VTS still suffered increased risk of poor perinatal outcomes such as LBW, jaundice of the newborn, and PTB. The later the fetus vanishes, the worse the perinatal outcomes would result. Based on the present findings, ultrasound examination was suggested in the first trimester in all women with twin pregnancies, especially in nulliparous or anovulatory ones, to screen for EVT. Those diagnosed with VTS, particularly LVT, should be taken care of and monitored closely during the perinatal period. However, as the solution from the origin, elective single-embryo transfer was suggested to mitigate the risk of multiple pregnancy.

## Data Availability Statement

The original contributions presented in the study are included in the article/supplementary material. Further inquiries can be directed to the corresponding authors.

## Ethics Statement

The studies involving human participants were reviewed and approved by the Reproductive Medicine Ethics Committee, Hospital for Reproductive Medicine Affiliated to Shandong University. Written informed consent to participate in this study was provided by the participants’ legal guardian/next of kin.

## Author Contributions

JrL performed the statistical analyses and wrote the manuscript. JyL checked the data, modified the article, and coordinated practical research assistance. YZ, KH, NC, JG, and Z-JC were involved in the sample collection and selection and phenotype data preparation. JH and LC are the guarantors of this work and, as such, had full access to all the data in the study and take responsibility for the integrity of the data and the accuracy of the data analysis. All authors contributed to the article and approved the submitted version.

## Funding

This study was supported by the National Key Research and Development Program of China (2018YFC1004301), Research Unit of Gametogenesis and Health of ART-Offspring, Chinese Academy of Medical Sciences (2020RU001), Shandong Provincial Key Research and Development Program (2018YFJH0504), Natural Science Foundation of Shandong Province of China (ZR2020MH065), Taishan Scholars Program for Young Experts of Shandong Province(tsqn201909195), and China Health Promotion Foundation (Association between ovarian response and embryo quality in women with normal ovarian reserve undergoing *in vitro* fertilization using a standard long protocol: a retrospective cohort study; Association between the numbers of retrieved oocytes and pregnancy outcomes in women with normal ovarian reserve undergoing *in vitro* fertilization: a retrospective cohort study).

## Conflict of Interest

The authors declare that the research was conducted in the absence of any commercial or financial relationships that could be construed as a potential conflict of interest.

## Publisher’s Note

All claims expressed in this article are solely those of the authors and do not necessarily represent those of their affiliated organizations, or those of the publisher, the editors and the reviewers. Any product that may be evaluated in this article, or claim that may be made by its manufacturer, is not guaranteed or endorsed by the publisher.
